# Philadelphia Hospital

**Published:** 1844-03-23

**Authors:** 


### CLINICAL LECTURES AND REPORTS.

### PHILADELPHIA HOSPITAL.

CLINIC OF THE JEFFERSON MEDICAL COLLEGE.

*February* 10, 1844.

BY PROFESSOR DUNGLISON.

[Reported by E. R. Squibb, and Samuel G. White.]

#### ANOMALOUS GASTRO-ENTERIC AFFECTION.

The case first presented to the class was very interesting on account of its singularity, and was introduced by the lecturer merely as a pathological curiosity—not as admitting of any special treatment. The patient, a man of middle age, is capable, by the action of his abdominal muscles, of producing at will a strange rattling or gurgling sound in the abdomen, which was quite audible to the class, even at some distance. The history derived from him was, that, about three years since, he had an attack of intermittent fever; following which, a painful tumour appeared in the left side, opposite the umbilicus.—Having augmented in size for about eighteen months, it suddenly disappeared; its recession being coincident with a discharge of bloody, purulent matter, *per anum;* since which time he has had the power of producing this singulai noise. The lecturer thought it probable, that, originally, owing to chronic sero-enteritis, a union had taken place between the stomach and colon, and that a collection of purulent matter had formed and been discharged into the intestines. The stomach now appears to be drawn down below its ordinary position, and to be enlarged, so that its liquid and gaseous contents can be agitated or churned together, by the voluntary action of the diaphragm and abdominal muscles.

#### PHARYNGITIS, ETC.

The next case was shown chiefly to illustrate the subject at present taught by the Professor from his chair in the College, viz.: the size and shape of the head as indicative of the intellectual sphere. The whole head was small for a man of the patient's stature; the forehead unusually retreating and narrow; and the anterior and superior part of the brain, consequently, much smaller than common. The facial expression and the restricted intellectual manifestations were in accordance with the defective encephalic organization.

The case was also instructive as exhibiting the confluent form of the pustular eruption produced by the tartrate of antimony and potassa, when applied to the skin; which, if seen for the first time, without a knowledge of the cause, might prove embarrassing in diagnosis to the young practitioner, and he ought, therefore, to be familiar with its appearance. The ointment had been applied to the neck for subacute pharyngitis, which had yielded under its use.

### HYDROPIC CACHEXIA.

The case of hydropic cachexia presented at the last clinic—in which diagnosis by exclusion had failed to detect any functional derangement to account for the effusion, and which was concluded to be as much a case of idiopathic dropsy as any that fall under attention, and therefore better adapted to exhibit the effects of the treatment proper to be instituted in such cases, than any of the others at present in the wards—was again exhibited to the class. Under the treatment then directed, the case is fast approaching a cure; the fluctuation in the abdomen and œdema of the extremities have entirely disappeared, and the manifest improvement in the patient's whole condition exhibits the propriety with which the remedies have been prescribed and administered.

The Professor remarked, that as an hydropic diathesis evidently exists in this case, it will be necessary to overcome the tendency to the re-accumulation of fluid by diuretics, combined with a tonic regimen, and the observance of strict sobriety on the part of the patient, whose habits had been previously irregular.

### AORTIC ANEURISM.

Modern observation shows, that the occurrence of internal aneurism is more frequent than was once supposed. There have been three cases in the wards of this Hospital, under the charge of the lecturer, since the commencement of the clinical year. He attributes this apparently greater frequency to the extreme difficulty in many cases in diagnosticating, especially prior to the introduction of the study of the physical signs, which afford us the strongest evidence, and often the only evidence, of the existence of the disease.

Internal aneurisms may be divided into five varieties.

First.—*Simple aneurism*, consisting of a simple general dilatation of the whole circumference of an arterial vessel.

Secondly.—*True aneurism*, in which, by the dilatation of the coats of a vessel in some circumscribed part, a sack or pouch is formed.

Thirdly.—*False aneurism*, when, by a rupture of the inner and middle coats, the cellular coat alone becomes distended.

Fourthly.—*Mixed aneurism*, in which the false becomes engrafted, as it were, upon the true, by a rupture of the inner and middle coats of the latter; and,

Fifthly,—*Dissecting aneurism*, produced by a giving way of the inner coat, whereby the blood separates the fibres of the middle coat, between which the blood forms itself a channel, and which, the lecturer said, had been well described by his friend and former colleague, Dr. Pennock; and, more recently, by Dr. Peacock, in the Edinburgh Medical and Surgical Journal for October, 1843.

In consequence of the greater pressure of the blood, aneurisms are most frequently formed in the ascending aorta, with the exception of the false, which being generally the result of mechanical violence, is most commonly seen in the descending. The Professor thinks that a morbid softening of the inner coats, especially, probably the result of *endo-aortitis*, always precedes the occurrence of aneurisms; acting either as the sole cause, or in concurrence with a diseased condition—as hypertrophy—of the heart.

In the diagnosis of these affections, the functional phenomena are most fallacious; none being present that may not be referred to other causes. It is upon the physical signs, taken along with the history of the case, that dependence alone can be placed. Thus the dyspnœa resulting from pressure of the tumour upon the air passages, might be owing to bronchitis or to pneumonia; and the tumefaction of the chest, to internal abscess, or to encephaloid degeneration of the lung, to which pulsation might be communicated by contact with the great vessels:—in short, none of the functional expressions afford any satisfactory basis for diagnosis: hence the necessity for extreme care in the discrimination; and, after all, dissection alone, at times, reveals the true nature of the case.

The prognosis in internal aneurism is always unfavourable, as the vessel will, in most cases, continue to expand, until rupture from ulceration or excessive tension causes the death of the patient from internal hæmorrhage. Cases of spontaneous cure are certainly rare.

Illustrative of these remarks, the Professor directed the attention of the class to cases at present in the wards; and, subsequently, to a marked pathological specimen from one which had just terminated fatally, of the history of which the following is an abstract:

Caroline W——, æt. 40, was admitted into the wards on the 26th of January. Her health had been feeble for some time, and she had suffered from palpitation and shortness of breath on making exertion. When admitted, she complained of acute pain in the left axilla, and expectorated mucus tinged with blood. A short time after this, she was seized with hæmatemesis, which occurred in the morning, followed by hæmoptysis in the evening,—transudations which indicated obstruction of the circulation. Pain and tenderness on pressure were experienced over the second rib of the right side, at its sternal junction. Percussion, on the right side, was dull throughout; on the left, there was slight dulness over the lower lobe: posteriorly the heart did not appear notably enlarged. On auscultation, the respiratory murmur could be heard over the entire left lung, mingled, however, with a sub-mucous *râle* at the posterior inferior part. Over the right, anteriorly, increased resonance of the voice; posteriorly, slight vesicular murmur was audible. The sounds of the heart were distinctly heard, the first being roughened. A tumour existed on the right side, near the sternum, over which the sounds of the heart and its pulsations could be distinguished, but in which there was no aneurismal thrill. She was treated by revellents, &c., for the pain in the axilla, under which slight improvement was evinced, until the night of January 31, when she was seized with excessive dyspnœa; increased pain; with manifest enlargement of the tumour. On the following morning, she first came under the Professor's notice, when he found the pulsation in the tumour obscure, and the sounds of the heart indefinable and indistinct,—a confused, rumbling noise only being heard. Although she was too feeble to admit of close examination, an aortic aneurism was presumed to exist. On the following day she became insensible, and remained in this state until the fourth of February, when she expired.

The necroscopy revealed the existence of an aneurismal tumour of the aorta, of the size of the head of the fœtus at term. This was found to be a true aneurism, and occupied nearly the whole of the right side of the chest; the lung being compressed, and the lower lobe almost entirely consolidated. At the bottom of the sac was found the orifice, through which blood had escaped into the cavity of the pleura. The heart was somewhat dilated, but not hypertrophied; the aorta was rough and lined by a deposite of ossific matter, some of the scales of which were as large as a quarter of a dollar; the lining membrane of the sac was also studded with ossific matter, proving its identity with the inner coat of the aorta; the orifice by which the aneurism communicated with the aorta, was one inch and a half in diameter. The sac was filled with dense coagula, separable into laminæ, from the outermost of which the red corpuscles had been almost entirely removed by absorption.

### PNEUMONITIC ABSCESS.

The attention of the class was lastly directed to another pathological specimen, presented by the dissection of the case of pneumonitic abscess which had been previously noticed by the Professor, (p. 28;) and which had passed through the various stages then prognosticated to its fatal termination.

The left lung presented, anteriorly, a very extensive superficial abscess, from which the patient had expectorated, at times, a pint of purulent matter per day. Tuberculous depositions had taken place in various parts of both lungs: and, at the top of the left, they had proceeded to the formation of a cavity. Both lungs were distinctly hypertrophied.

